# Aboveground biomass estimation of wetland vegetation at the species level using unoccupied aerial vehicle RGB imagery

**DOI:** 10.3389/fpls.2023.1181887

**Published:** 2023-07-17

**Authors:** Rui Zhou, Chao Yang, Enhua Li, Xiaobin Cai, Xuelei Wang

**Affiliations:** ^1^Key Laboratory for Environment and Disaster Monitoring and Evaluation of Hubei, Innovation Academy for Precision Measurement Science and Technology, Chinese Academy of Sciences, Wuhan, China; ^2^University of Chinese Academy of Sciences, Beijing, China; ^3^Honghu Lake Station for Wetland Ecosystem Research, Chinese Academy of Sciences, Honghu, China; ^4^Yangtze River Basin Ecological Environment Monitoring and Scientific Research Center, Yangtze River Basin Ecological Environment Supervision and Administration Bureau, Ministry of Ecological Environment, Wuhan, China

**Keywords:** wetland vegetation, *Zizania latifolia*, aboveground biomass, unoccupied aerial vehicle, back propagation neural network

## Abstract

Wetland vegetation biomass is an essential indicator of wetland health, and its estimation has become an active area of research. *Zizania latifolia* (*Z. latifolia*) is the dominant species of emergent vegetation in Honghu Wetland, and monitoring its aboveground biomass (AGB) can provide a scientific basis for the protection and restoration of this and other wetlands along the Yangtze River. This study aimed to develop a method for the AGB estimation of *Z. latifolia* in Honghu Wetland using high-resolution RGB imagery acquired from an unoccupied aerial vehicle (UAV). The spatial distribution of *Z. latifolia* was first extracted through an object-based classification method using the field survey data and UAV RGB imagery. Linear, quadratic, exponential and back propagation neural network (BPNN) models were constructed based on 17 vegetation indices calculated from RGB images to invert the AGB. The results showed that: (1) The visible vegetation indices were significantly correlated with the AGB of *Z. latifolia*. The absolute value of the correlation coefficient between the AGB and CIVE was 0.87, followed by ExG (0.866) and COM2 (0.837). (2) Among the linear, quadratic, and exponential models, the quadric model based on CIVE had the highest inversion accuracy, with a validation R^2^ of 0.37, RMSE and MAE of 853.76 g/m^2^ and 671.28 g/m^2^, respectively. (3) The BPNN model constructed with eight factors correlated with the AGB had the best inversion effect, with a validation R^2^ of 0.68, RMSE and MAE of 732.88 g/m^2^ and 583.18 g/m^2^, respectively. ​Compared to the quadratic model constructed by CIVE, the BPNN model achieved better results, with a reduction of 120.88 g/m^2^ in RMSE and 88.10 g/m^2^ in MAE. This study indicates that using UAV-based RGB images and the BPNN model provides an effective and accurate technique for the AGB estimation of dominant wetland species, making it possible to efficiently and dynamically monitor wetland vegetation cost-effectively.

## 1 Introduction

Wetland vegetation is an important component of wetland ecosystems and plays a crucial role in the ecological function of the wetland environment (Wan et al., 2019; Zhou et al., 2021). Aboveground biomass (AGB) of wetland vegetation serves as a key indicator to evaluate the health status and the carbon storage capacity of wetland ecosystems (Shen et al., 2021). Monitoring the AGB of wetland vegetation can provide a scientific basis for the conservation and restoration of wetland ecosystems, which is essential for achieving carbon neutrality targets (Shao et al., 2022). Due to the poor accessibility of wetlands and the influence of complex environmental factors, traditional manual harvesting methods for obtaining the AGB is not only time-consuming and labor-intensive, but also difficult to implement on a large scale.

Remote sensing techniques that provide timely, up-to-date spatial information are increasingly indispensable for wetland assessment and management, overcoming the limitations of traditional approaches (Adam et al., 2010). As an emerging low-altitude remote sensing technology, unoccupied aerial vehicles (UAVs) are more convenient platforms for remote sensing data acquisition than satellites. UAV remote sensing not only has the advantage of high flexibility and cost efficiency, but also high spatial resolution from sub-meter to centimeter level, providing high spatial detail (Klemas, 2015). Therefore, the use of UAV-based images has the potential to explain the heterogeneous structure of wetland vegetation (Jing et al., 2017). Many studies have successfully performed wetland vegetation mapping and monitoring based on UAV remote sensing (Meng et al., 2017; Corti Meneses et al., 2018; Yan et al., 2019; Durgan et al., 2020; Zhou et al., 2021). Cao et al. (Cao et al., 2018) verified the effectiveness of UAV hyperspectral images in mangrove species identification. Fu et al. (Fu et al., 2021) evaluated the ability of the optimized Random Forest (RF) algorithm and SegNet algorithm to classify wetland vegetation communities based on low-altitude UAV images. In addition, the spatiotemporal monitoring of invasive species in wetlands has also been successfully conducted by remote sensing data derived from UAVs (Abeysinghe et al., 2019; Anderson et al., 2021; Brooks et al., 2021). Furthermore, efforts have also been paid to estimate wetland vegetation density and fractional vegetation cover (FVC) based on UAVs (Zhou et al., 2018; Pinton et al., 2021).

However, most of these studies are related to the horizonal surface information of wetland vegetation (Wang et al., 2017a), whereas there are far fewer studies on the AGB inversion of wetland vegetation than for forest (Lin et al., 2018), grassland (Villoslada Peciña et al., 2021) and crop biomass inversions (Zheng et al., 2019; Zhang et al., 2021). This is because the growing conditions of wetland vegetation are more complex with higher spatial and temporal variability compared to other types of ecosystems. In addition, due to the poor accessibility of wetlands, it is difficult to collect validation samples, hindering the development of wetland vegetation biomass studies. Doughty et al. successfully estimated marsh biomass by using the correlation between several vegetation indices and the AGB with a determination coefficient (R^2^) of 0.67 and a root mean square error (RMSE) of 344 g/m^2^ based on multispectral UAV imagery (Doughty and Cavanaugh, 2019). Their subsequent research results showed that the accuracy of UAV-based biomass inversion (R^2 ^= 0.40, RMSE=534.6 g/m^2^) was higher than the Landsat-based result (R^2 ^= 0.26, RMSE=596.8 g/m^2^), showing that UAV images could better reflect the spatial variability of wetland vegetation biomass at a fine scale (Doughty et al., 2021).

With the development of UAV technology, small consumer UAVs have been shown to be suitable for wetland surveys, overcoming the limitations of wetland vegetation biomass monitoring. Some scholars have pointed out that UAVs equipped with RGB sensors make the acquisition of high-resolution images and field survey data more convenient and affordable than UAVs equipped with other types of sensors (Kutugata et al., 2021). Furthermore, the visible vegetation indices generated from RGB images (e.g., Excess green index (ExG), Color index of vegetation (CIVE), Vegetation index (VEG), Combination index (COM)) have been shown to have a certain relationship with the growth status of vegetation, which is helpful for the quantitative and continuous vegetation monitoring (Klemas, 2015; Yue et al., 2019; Liu et al., 2021). Linear/nonlinear statistical regression models constructed by vegetation indices and field survey samples are the most commonly used for traditional vegetation biomass inversion (Bendig et al., 2015; Zheng et al., 2019). In recent years, studies have found that machine learning algorithms (e.g., random forest (RF), support vector machine (SVM), back propagation neural network (BPNN)) can perform better for vegetation biomass estimation due to the superior ability to identify and simulate the correlation of the datasets (Wang et al., 2016; Viljanen et al., 2018; Zhu et al., 2019; Sharma et al., 2022; Zhang et al., 2022). Presently, most existing studies about wetland vegetation have been conducted on landscape and community levels (Gao et al., 2019; Wan et al., 2019; Wang et al., 2022). Few researchers have attempted to combine machine learning algorithms and high-resolution RGB images to estimate the AGB of wetland vegetation at the species level.

The objective of this study is to demonstrate the feasibility of using high-resolution UAV RGB images for species-level AGB estimation in wetlands. *Zizania latifolia* (*Z. latifolia*), the dominant species of emergent vegetation in Honghu Wetland Nature Reserve, was selected as the research object to demonstrate the feasibility of UAV RGB images in the AGB estimation. *Z. latifolia*, commonly thriving in shallow water, exhibits robust growth. ​Its roots are firmly anchored in the waterbed, while the stem and leaves extend above the surface of the water. This article considers the fresh weight of the portion above the water surface as the AGB of *Z. latifolia*. First, we extracted multi-features from RGB images to map the spatial distribution of *Z. latifolia* using the object-based classification method. Then, the linear, quadratic, exponential regressions and the BPNN model were constructed based on 17 vegetation indices to invert the AGB of *Z. latifolia*. Finally, the accuracy of different inversion models was compared to figure out the optimal inversion model. This paper provides a technical reference for accurate and rapid AGB monitoring of wetland vegetation at the species level in an accurate and rapid way.

## 2 Materials

### 2.1 Study area

**Figure 1:**
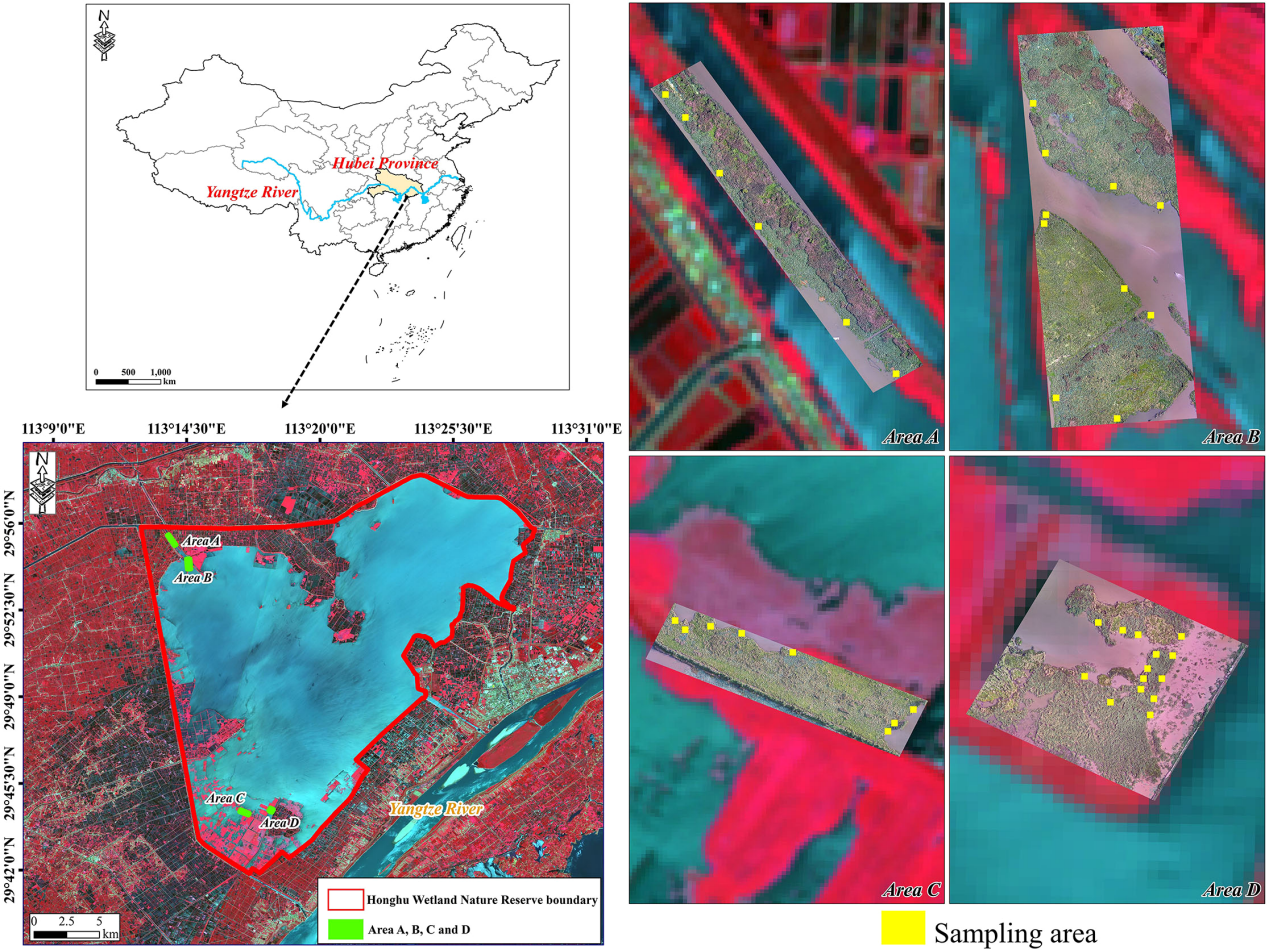
Study area in Honghu Wetland.

Honghu Wetland (29°41′–29°58′N, 113°12′–113°28′E), listed as a Wetland of International Importance under the Ramsar Convention, is located in the southeast of Hubei Province, China, with a surface area of approximately 414 km^2^ (**Figure 1**). The lowest average temperature in January is +3.8 °C, while the highest average temperature in July is +28.9 °C. Annual rainfall ranges from 1000 to 1300 mm. As the largest freshwater lake in Hubei Province, Honghu plays an important role in flood control and storage, water conservation, and biodiversity maintenance in the middle and lower reaches of the Yangtze River (Li et al., 2021). Honghu Wetland has excellent hydrological conditions suitable for vegetation growth and therefore high biodiversity. *Z. latifolia* is the dominant species of emergent vegetation in Honghu Wetland. Monitoring *Z. latifolia* is crucial to understanding the processes and laws of the carbon cycle in the Honghu Wetland ecosystem.

### 2.2 Acquisition of UAV-based RGB images

Based on the experience of field surveys conducted over several consecutive months, we selected four plots within the study area, namely Area A, B, C and D, for UAV-based RGB image acquisition. The four sample areas are located in the riparian zone and have rich vegetation types, which can typically represent the growth status of *Z. latifolia* in Honghu Wetland. The UAV flight mission was deployed on July 29 and 30, 2021, when *Z. latifolia* reached its peaking growing season. All the flights were conducted from 10:00 to 12:00 in cloudless and windless weather conditions. The original RGB images were acquired by the L1D-20c camera on a DJI Mavic Pro drone at an altitude of 100 m, with a lateral overlap of 70% and a forward overlap of 80%. The exposure and shutter speed were set depending on the light conditions. All images were imported to Pix4Dmapper to generate digital orthophoto maps (DOM) and digital surface models (DSM) for four plots. The final spatial resolution of the acquired RGB images was approximately 2.4 cm.

### 2.3 Field sampling data

We carried out field surveys simultaneously with UAV missions in four areas. Each plot had a large area of *Z. latifolia*, with vigorous growth stages and an average height of 1.20 to 1.54 m. A total of 38 sampling areas (0.6 m × .6 m) containing only *Z. latifolia* were randomly distributed in four areas (**Figure 1**). The geographical coordinates and average height of each sampling area were recorded. The *Z. latifolia* above the water level was harvested, and the AGB was measured using an electronic scale with an accuracy of 10 g. Out of the 38 field samples, 30 samples were selected randomly for modeling, and the remaining 8 samples were used for model validation.

## 3 Methods

**Figure 2:**
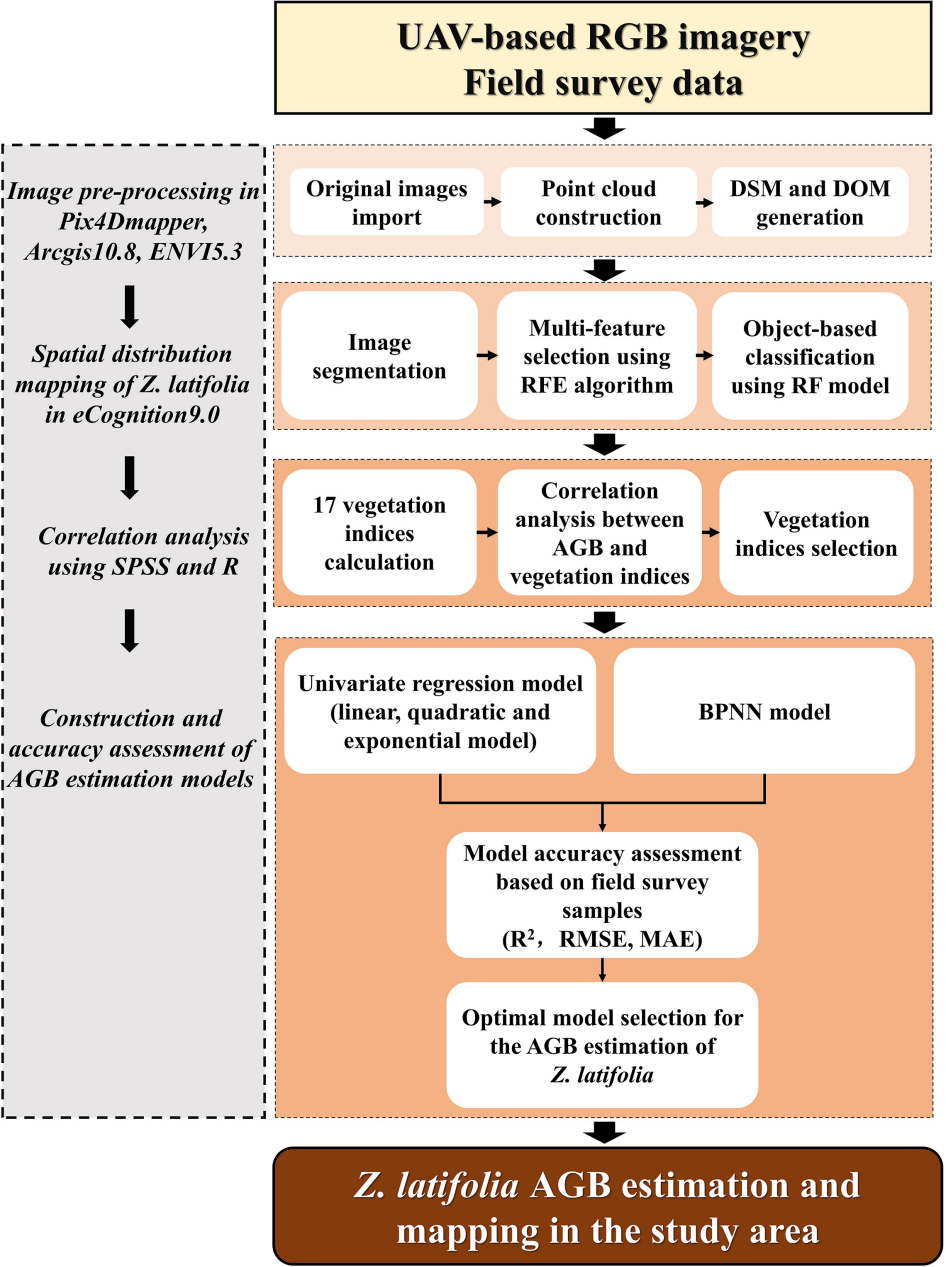
Workflow for the AGB estimation in this study.

In this study, the UAV-based RGB images and field survey data were used to estimate the AGB of *Z. latifolia* in Honghu Wetland. As shown in **Figure 2**, there were four main steps, including image pre-processing, spatial distribution mapping of *Z. latifolia*, correlation analysis between vegetation indices and the AGB, construction and accuracy assessment of AGB estimation models.

### 3.1 The object-based classification method

The object-based classification method uses the objects aggregated by pixels with minimal heterogeneity for classification, which is different from the traditional pixel-based classification method. Semantic features of objects derived from high-resolution images, such as texture, shape, topology and context, are used as inputs for machine learning algorithms to distinguish feature categories (Chen et al., 2018). Previous studies have reported that reducing the number of high-dimensional features is crucial for enhancing classifier performance, and the recursive feature elimination (RFE) algorithm is a commonly used feature selection method (Fu et al., 2022). Our previous study has also demonstrated that the combination of multi-feature selection (using the RFE) and object-based classification method can significantly improve the classification accuracy of wetland vegetation, with an overall accuracy exceeding 90% and the kappa above 0.9, and user’s accuracy (UA) and producer’s accuracy (PA) of *Z. latifolia* were 75% and 95.45% (Zhou et al., 2021). This accuracy is considered to meet the follow-up requirements for the AGB estimation of *Z. latifolia*.

In the present study, we first extracted a total of 53 features including spectral information, texture features, vegetation indices, height information and geometric features in eCognition Developer 9.0. Then, the RFE was used for multi-feature selection to remove redundant features in RStudio. The selected features were set as the input of the RF model for *Z. latifolia* classification. Finally, the spatial distribution of *Z. latifolia* in four areas was obtained through this semi-automated classification process.

### 3.2 Selection of visible vegetation indices

A visible vegetation index is formed by the combination of R, G and B bands, which effectively reflects the changes in vegetation canopy spectral information, and is widely used in vegetation classification and biomass estimation (Zhang et al., 2019). Based on existing research results, 17 commonly used visible light vegetation indices were extracted from UAV-based RGB images, including R, G, B, Red–green ratio index (RGRI), Blue–green ratio index (BGRI), Woebbecke index (WI), Normalized green–red difference index (NGRDI), Normalized green–blue difference index (NGBDI), Red–green–blue ratio index (RGBRI), Vegetation index (VEG), Color index of vegetation (CIVE), Excess green index (ExG), Excess green minus excess red index (ExGR), Combination index (COM), Combination index 2 (COM2), Visible-band difference vegetation index (VDVI) and Red–green–blue vegetation (RGBVI) (Woebbecke et al., 1995; Guijarro et al., 2011; Guerrero et al., 2012; Calderon et al., 2013; Torres-Sanchez et al., 2014; Bendig et al., 2015; Du and Noguchi, 2017; Wan et al., 2018; Xie et al., 2020). The description of each vegetation index is shown in **Table 1**. To avoid data redundancy by using all the vegetation indices for modeling, Pearson Correlation Analysis was used to study the correlation between the AGB of *Z. latifolia* and vegetation indices, and the main determinants of the biomass were determined. In this study, the ground resolution of the UAV-RGB images in four areas was resampled to 0.6 m to keep it consistent with the size of the field samples. Centering on each sample point, we calculated the average of the 5 × pixels as the value of each vegetation index. Then, IBM SPSS Statistics 25 and RStudio software were used to execute correlation analysis and visualization.

**Table 1:** Vegetation indices based on UAV RGB images.

**Vegetation Indices**	**Calculation Formula**
R	reflectance values of the red band
G	reflectance values of the green band
B	reflectance values of the blue band
Red–green ratio index (RGRI)	R/G
Blue–green ratio index (BGRI)	B/G
Woebbecke index (WI)	(G−B)/(R−G)
Normalized green–red difference index (NGRDI)	(G−R)/(G+R)
Normalized green–blue difference index (NGBDI)	(G−B)/(G+B)
Red–green–blue ratio index (RGBRI)	(R+B)/2G
Vegetation index (VEG)	$G/R^{0.67}B^{0.33}$
Color index of vegetation (CIVE)	0.44R−0.88G+0.39B+18.79
Excess green index (EXG)	2G−R−B
Excess green minus excess red index (ExGR)	ExG−1.4R−G
Combination index (COM)	0.25ExG+0.3ExGR+0.33CIVE+0.12VEG
Combination index 2 (COM2)	0.36ExG+0.47CIVE+0.17VEG
Visible-band difference vegetation index (VDVI)	(2G−R−B)/(2G+R+B)
Red–green–blue vegetation (RGBVI)	$(G^{2}-\left(R*B\right))/\left(G^{2}+\left(R*B\right)\right)$

### 3.3 Construction of AGB estimation models

#### 3.3.1 Univariate regression analyses

Regression analysis modeling is used to study the quantitative relationship between different variables by establishing mathematical models and is widely applied in biomass inversion (Gao et al., 2017). In this paper, the AGB of *Z. latifolia* was taken as the dependent variable, and vegetation indices were taken as independent variables. The linear, quadratic and exponential models with single variable were constructed, and the accuracy of each regression model was compared to obtain the optimal regression model for the AGB inversion of *Z. latifolia*.

#### 3.3.2 Artificial neural network model

**Figure 3:**
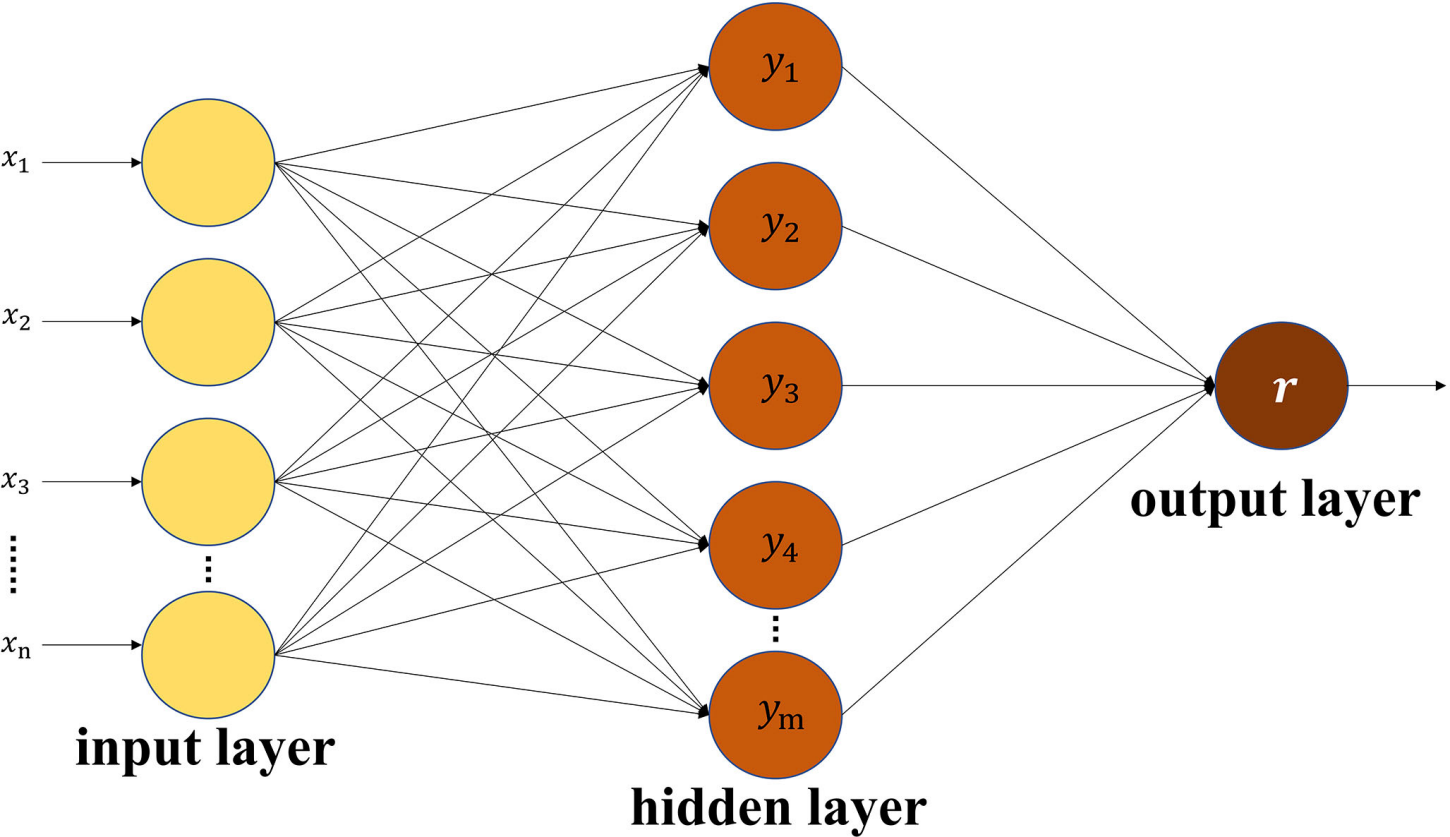
Structure of the back propagation neural network.

The artificial neural network model can better simulate the nonlinear relationship between each variable, reduce the error caused by human intervention, and is more practical than other linear or nonlinear regression models (Deb et al., 2017; Yang et al., 2018). The BPNN is one of the most widely used neural network models with obvious advantages for complex data processing. It approximates the original law of the data mainly through repeated training and continuous fitting (Han et al., 2018). The BPNN is a multi-layer feedforward network trained by an error back propagation algorithm, which mainly includes two processes: information forward propagation and error back propagation. A three-layer BP neural network model is composed of an input layer, a hidden layer and an output layer (Yang et al., 2018). As shown in **Figure 3**, n is the number of input neurons, m is the number of hidden neurons, and r is the output neuron of the output layer (Wang et al., 2019). The number of neurons in the hidden layer needs to be determined through experience and repetitive experiments, which affects the effect of model fitting. The basic idea of the BPNN is to input samples to the hidden layer for processing and then transmit it to the output layer. If the error is large, it will carry out back propagation, and reduce the error by modifying the number of neurons until the expected value is reached, and then the network training is completed.

In this study, the BPNN model was constructed using the neural network toolbox in Matlab software. Vegetation indices strongly correlated with the AGB of *Z. latifolia* were used as the input of the BPNN model, and the parameters were continuously adjusted until the constructed model met the accuracy requirements.

### 3.4 Model accuracy assessment

The mean absolute error (MAE), RMSE and R^2^ were used to evaluate the performance of each model. MAE represents the average value of the absolute errors. RMSE is used to measure the deviation between the predicted value and the measured value. R^2^ is a commonly used index to judge the fitting effect of a model with a value between 0 and 1. A model with a higher R^2^, a smaller MAE and RMSE owns higher AGB inversion accuracy (Yue et al., 2019). The calculation formula is shown as follows (Huang et al., 2016):


(1) $$\mathrm{MAE}=\sum_{i=1}^{n}\left|\left({\text{Y}}_{i}-{\text{y}}_{i}\;\right)\right|/\text{n}$$

(2) $$\mathrm{RMSE}=\sqrt{\sum_{i=1}^{n}({\text{Y}}_{i}-{\text{y}}_{i}{)}^{2}/\text{n}}$$

(3) $${\text{R}}^{2}=1-[\sum_{i=1}^{n}{\left({\text{Y}}_{i}-{\text{y}}_{i}\right)}^{2}/\sum_{i=1}^{n}{\left({\text{Y}}_{i}-\bar{\text{y}}\right)}^{2}]$$

where $Y_{i}$ is the measured AGB of sample i, $y_{i}$ is the estimated AGB of sample i, $\bar{y\;}$ the estimated mean AGB, n is the number of validation samples.

## 4 Results

### 4.1 Spatial distribution mapping of *Z. latifolia*

**Figure 4:**
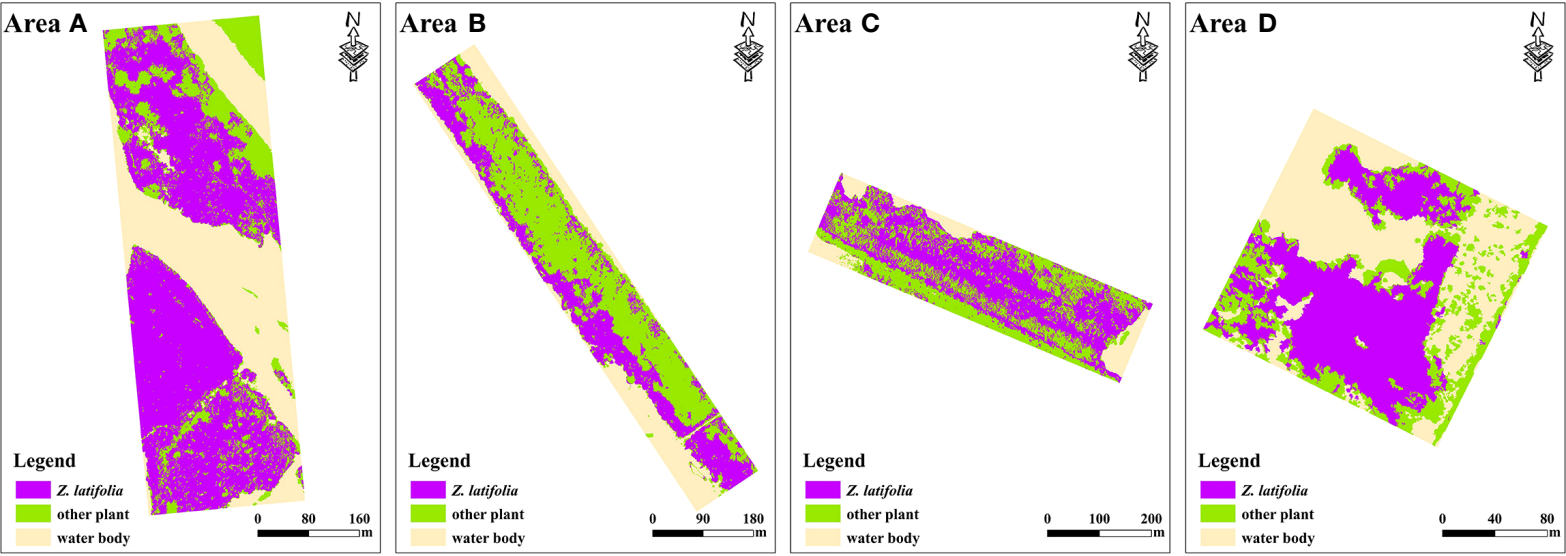
Spatial distribution mapping of *Z. latifolia*.

According to the features derived from the UAV-RGB images, the spatial information of *Z. latifolia* in area A-D was extracted (**Figure 4**). Based on field surveys, we selected 231 ground validation samples to evaluate the classification accuracy of *Z. latifolia*. The validation results showed that the overall accuracy was more than 90.7%. *Z. latifolia* had fewer misclassifications and omission classifications, with both the PA and UA exceeding 90%, which was considered to meet the experimental requirements of subsequent AGB inversion.

### 4.2 Correlation analysis

**Figure 5:**
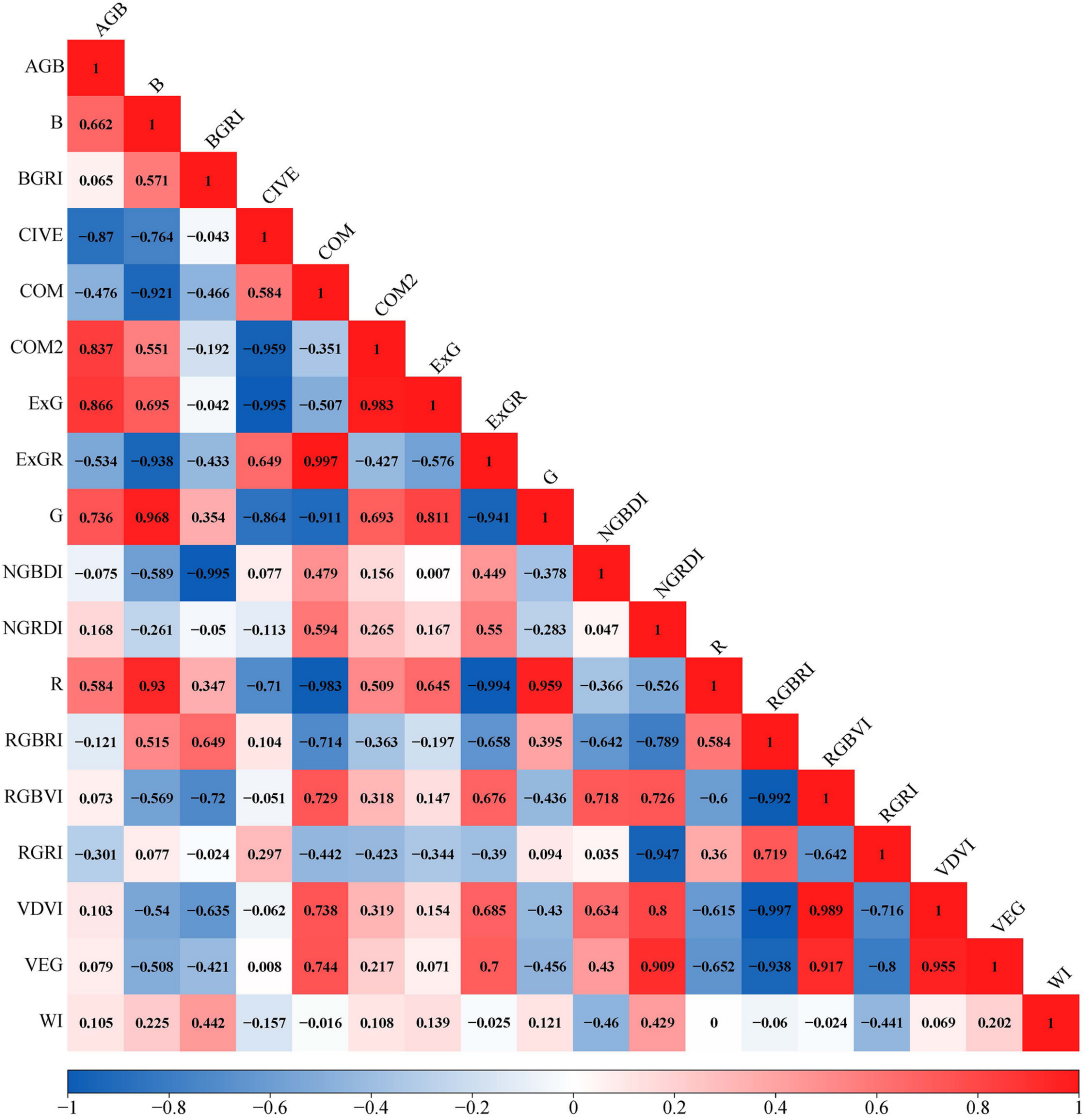
Visualization of the correlation matrix for vegetation indices and the AGB. Red indicates positive correlation, blue indicates negative correlation, and the darker the color, the stronger the correlation.

The visualization result of the correlation matrix showed that eight of seventeen vegetation indices were significantly correlated with the AGB of *Z. latifolia* (**Figure 5**). We discovered that the measured AGB of *Z. latifolia* had a strong negative correlation with CIVE (-0.870), and a strong positive correlation with ExG (0.866) and COM2 (0.837). In addition, the AGB was also positively correlated with G (0.736), B (0.662) and R (0.584), and negatively correlated with ExGR (-0.534) and COM (-0.476). The remaining nine of seventeen vegetation indices were not correlated with the AGB of *Z. latifolia*.

### 4.3 Accuracy assessment of univariate regression models

**Figure 6:**
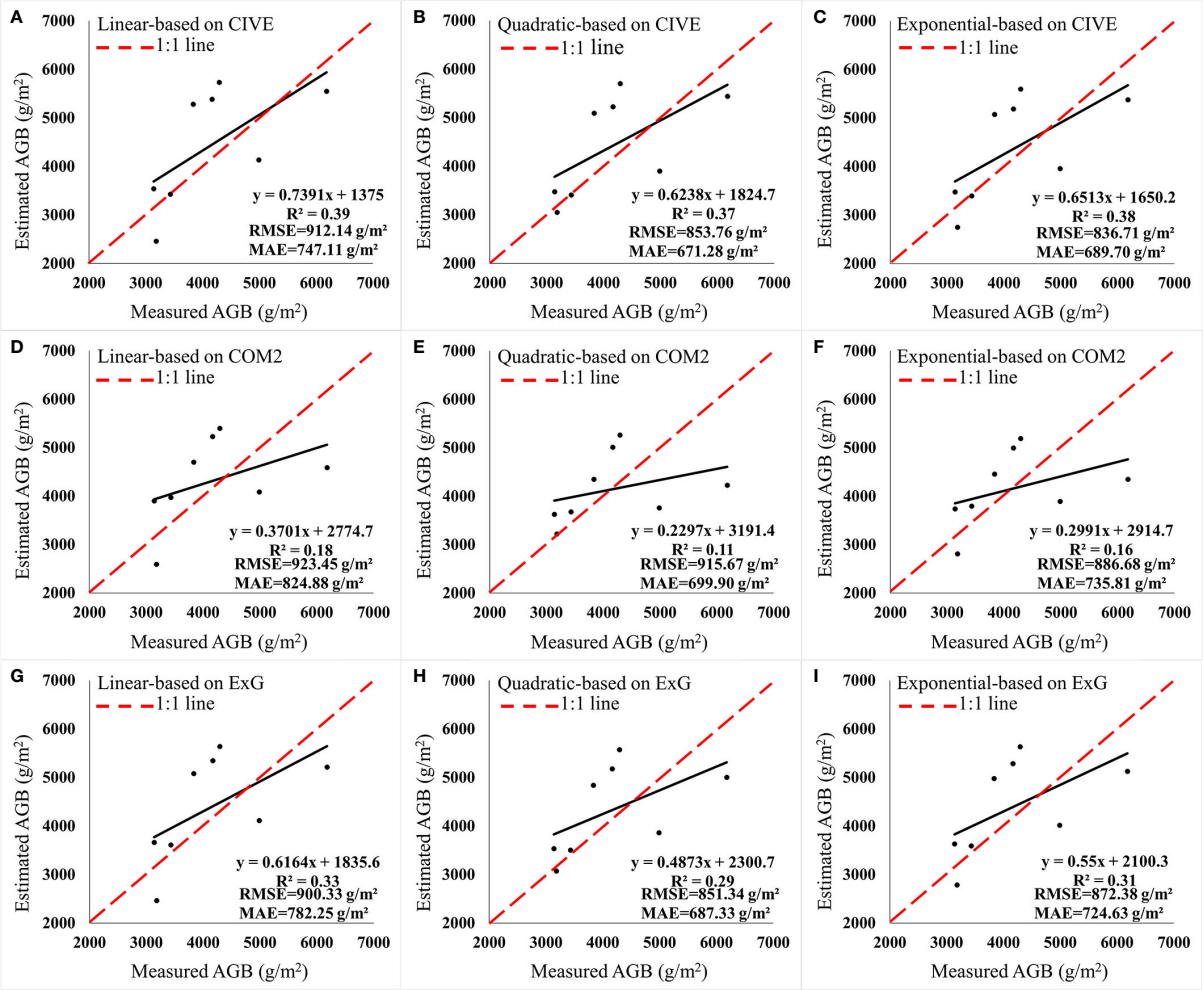
Accuracy validation of univariate regression models. **(A–C)** Models based on CIVE. **(D–F)** Models based on COM2. **(G–I)** Models based on ExG.

According to the correlation analysis, vegetation indices with an absolute correlation greater than 0.8 (CIVE, ExG and COM2) were selected to construct univariate regression models (**Table 2**). For different vegetation indices, the modeling R^2^ of the quadratic model were all greater than or equal to 0.75 and were higher than those of the linear and exponential models. The quadratic model constructed by CIVE had the highest modeling R^2^ (0.79) which was higher than that of the quadratic model constructed by ExG (0.78) and COM2 (0.75). According to the accuracy validation results in **Figure 6**, CIVE was the best vegetation index for the AGB inversion of *Z. latifolia*. The quadratic model constructed by CIVE had the optimal inversion accuracy (validation R^2 ^= 0.37, RMSE=853.76 g/m^2^ and MAE=671.28 g/m^2^), followed by the quadratic model constructed by ExG (validation R^2 ^= 0.29, RMSE=851.34 g/m^2^ and MAE=687.33 g/m^2^).

**Table 2:** Construction of univariate regression models.

VIs	Regression method	Modeling equation	modeling R²	Validation equation
CIVE	Linear	y=-175.307x+2248.548	0.75	y=0.7391x+1375
	Quadratic	y=5.812x^2^-19.178x+3018.857	0.79	y=0.6238x+1824.7
	Exponential	y=2627.791exp(-0.038x)	0.74	y=0.6513x+1650.2
COM2	Linear	y=622.104x-6175.956	0.69	y=0.3701x+2774.7
	Quadratic	y=107.315x^2^-3058.055x+25007.853	0.75	y=0.2297x+3191.4
	Exponential	y=413.174exp (0.136x)	0.69	y=0.2991x+2914.7
ExG	Linear	y=85.613x-955.545	0.74	y=0.6164x+1835.6
	Quadratic	y=1.498x^2^-107.818x+4993.041	0.78	y=0.4837x+2300.7
	Exponential	y=1303.737exp (0.019x)	0.74	y=0.55x+2100.3

### 4.4 Accuracy assessment of the BPNN model

**Figure 7:**
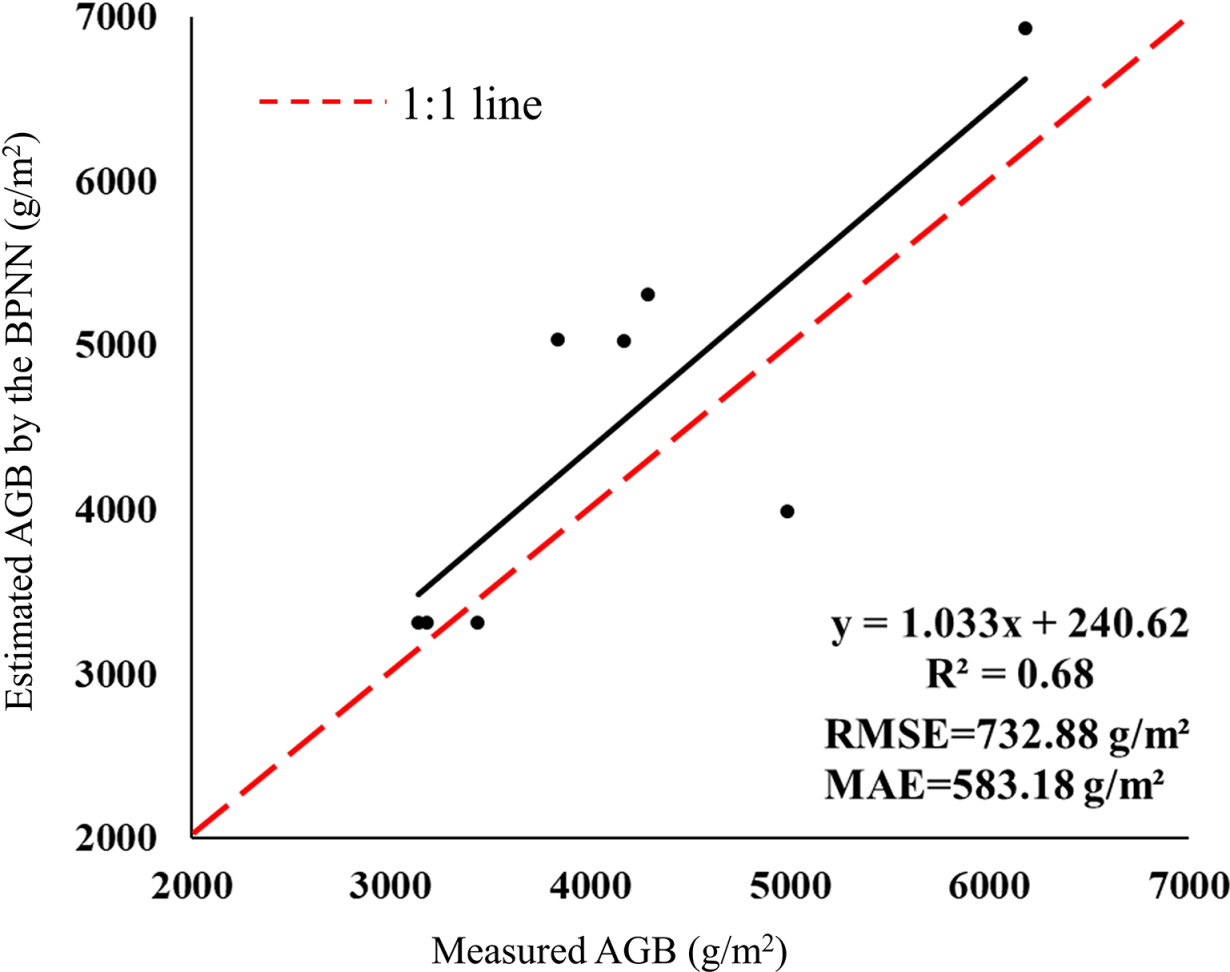
Accuracy validation of the BPNN model.

The R, G, B, ExG, ExGR, CIVE, COM and COM2, which are significantly correlated with the AGB of *Z. latifolia*, were selected as model inputs and the measured AGB was set as the model output. The range of the neuron numbers in the hidden layer was calculated based on a previously published formula, and then the optimal neuron number was determined through repeated experiments (Guo et al., 2000). Finally, the optimal neuron number in the hidden layer was set as three. The Levenberg-Marquardt algorithm (trainlm) was selected as the training method, and the maximum iteration was set as 1000. After continuous training, the final BPNN constructed met the accuracy requirements with a validation R^2^ of 0.68, RMSE and MAE of 732.88 g/m^2^ and 583.18 g/m^2^, respectively (**Figure 7**). Compared with the quadratic model constructed by CIVE, the validation R^2^ of the BPNN was increased by 0.31, RMSE and MAE were reduced by 120.88 g/m^2^ and 88.10 g/m^2^, respectively. The results showed that the inversion accuracy of the BPNN model was significantly improved compared with univariate models, indicating that the BPNN model could effectively improve the AGB inversion accuracy of *Z. latifolia*.

**Figure 8:**
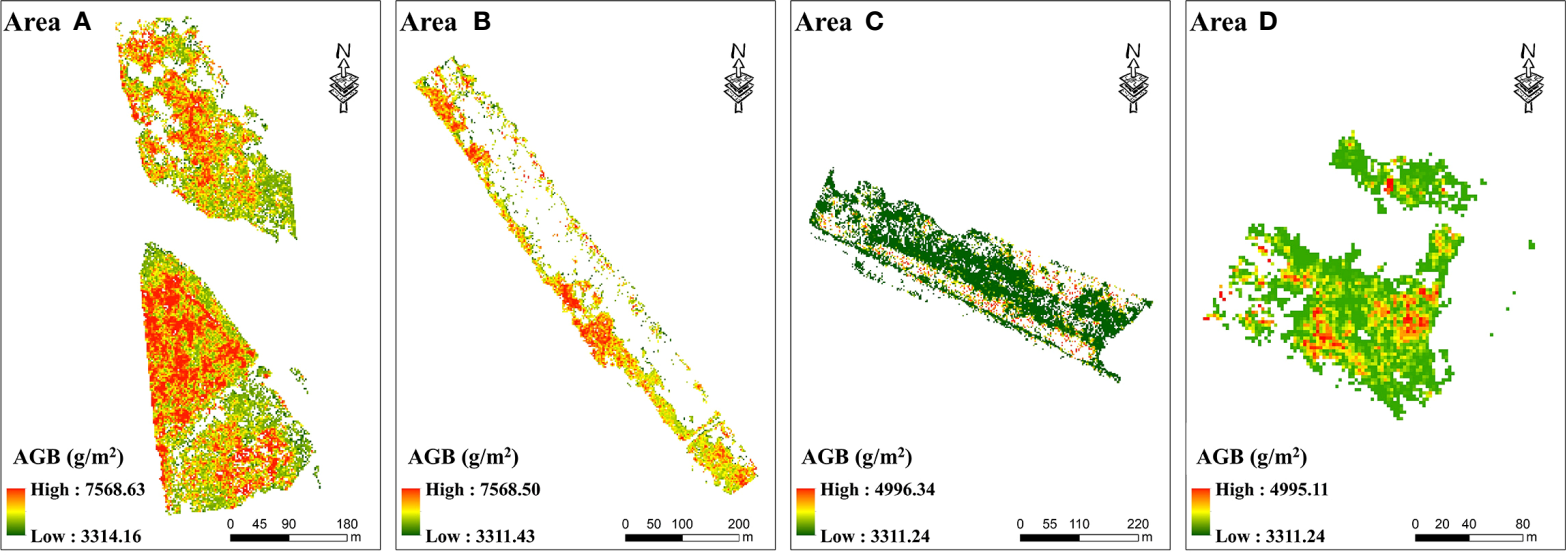
Spatial distribution of the AGB of *Z. latifolia* inversed by the BPNN model.

The BPNN model satisfying the inversion accuracy requirements was used to simulate the AGB of *Z. latifolia* in Area A, B, C and D. As shown in **Figure 8**, the highest AGB of *Z. latifolia* was about 7568 g/m^2^ in Area A and B, and about 4996 g/m^2^ in Area C and D. There was no significant difference in the lowest AGB of four areas, which was about 3311 g/m^2^. Area B had the highest average AGB at 6132.57 g/m^2^, while Area A had a slightly lower average AGB at 5879.54 g/m^2^. The average AGB of Area C and Area D was 3499.97 g/m^2^ and 3653.96 g/m^2^, respectively, which was lower than Area A and B.

## 5 Discussion

### 5.1 Correlations between AGB and vegetation indices

In the present study, the AGB of *Z. latifolia* was positively correlated with ExG, COM2, R, G, and B, and negatively correlated with ExGR, CIVE, and COM. ​Our results confirmed that CIVE, COM2, and ExG were the most important metrics for the AGB inversion of *Z. latifolia*, with the a correlation coefficient above 0.8 in absolute value, which is consistent with previous research results (Zhang et al., 2018; Kutugata et al., 2021; Anchal et al., 2022; Chen et al., 2022). According to the results of the correlation visualization, there is a high correlation between vegetation indices. Only WI had a poor correlation with other vegetation indices, and Morgan et al. (Morgan et al., 2021) also had similar results in their previous study. Vegetation indices are closely related to vegetation growth. The visible vegetation indices derived from high-resolution RGB images make it possible to quickly and economically monitor wetland vegetation biomass. Due to the different characteristics of wetland vegetation, the construction and selection of vegetation indices is important to improve the accuracy of biomass estimation.

### 5.2 Advantages and disadvantages of BPNN models

Compared with the traditional univariate regression models, BPNN models have strong advantages in solving complex non-linear data, enhancing data processing efficiency and improving the AGB estimation accuracy. Numerous studies have confirmed the usefulness of BPNN models for vegetation classification, grass biomass inversion, and vegetation growth monitoring, but their superiority in wetland vegetation biomass estimation has been less elaborated (Zhu et al., 2015; Yang et al., 2018; Wang et al., 2019). In this study, the BPNN model constructed by the vegetation indices that were significantly correlated with the AGB of *Z. latifolia* achieved high estimation accuracy with a validation R^2^ of 0.68, RMSE and MAE of 732.88 g/m^2^ and 583.18 g/m^2^, respectively. The estimation accuracy of the BPNN model was significantly higher than that of traditional regression models, which is consistent with the results of previous studies (Yang et al., 2018; Zhu et al., 2019). Therefore, BPNN models hold potential in the monitoring and mapping the biomass of wetland vegetation, but still suffer from some disadvantages such as slow convergence speed and being easily trapped in local minima (Wang et al., 2017b; Gao et al., 2018; Wang et al., 2019). The commonly used machine learning algorithms such as RF, SVM, etc., have specific advantages and disadvantages. Whether the combination of multiple machine learning algorithms can effectively improve the inversion accuracy of biomass of wetland vegetation in subsequent studies needs to be further explored.

### 5.3 AGB estimation by using UAV-based RGB imagery

Due to the complexity of wetland environments, monitoring wetland vegetation at the species level has been studied poorly. However, the biomass mapping of dominant species in wetlands is ecologically important. In this study, we have achieved high accuracy in the AGB inversion, which can provide a reference for monitoring *Z. latifolia*, as well as other dominant species in wetlands. We demonstrated that high-resolution UAV-based RGB imagery can bridge the gap between field survey data and remote sensing data, making it possible to map species-level biomass. This is consistent with other studies that UAVs as an emerging low-altitude remote sensing technology can provide cost-effective manners for surveying and monitoring resources, such as barley biomass estimation, tree count derivation, and forest biomass estimation (Bendig et al., 2015; Mohan et al., 2017). Our previous study showed that high-resolution UAV-based RGB imagery enables fine classifications of wetland vegetation, laying the foundation for subsequent biomass inversion of individual species (Zhou et al., 2021). Lopatin et al. (Lopatin and Lopatina, 2017) also successfully conducted a similar study using UAV imagery to map the biomass distribution of *Phragmites australis*, and pointed out that UAV remote sensing has great potential to provide accurate maps of biomass distribution at different phenological stages.

In this study, despite providing an effective technical method for the AGB estimation of wetland vegetation at the species level, there are still some limitations. First, wetland environments are complex with low accessibility, and the limited number of field samples affects the generalization ability of the BPNN model and reduces the estimation accuracy. Second, it is difficult to implement biomass inversion on a large scale due to the limited coverage area of UAV images. Based on current research status, further studies can explore the use of non-destructive indicators acquired from UAV imagery to invert wetland vegetation biomass, such as Fractional Vegetation Cover (FVC), which can not only avoid the difficulties of sample collection, but also reduces the damage to the wetland environment caused by field sampling. Similar studies have been done to demonstrate that there is a correlation between FVC and the biomass of shrub communities (Guo et al., 2021). However, the applicability in the estimation of wetland vegetation biomass remains to be verified. In addition, due to the convenience and timeliness of consumer UAVs, we can construct a field sample library for vegetations at different phenological stages in the expectation of achieving automatic matching of vegetation biomass. Although using the visible vegetation indices for the AGB estimation can yield satisfactory results, the influence of the introduction of texture features generated by UAV high-resolution images on the estimation accuracy remains to be explored. Furthermore, the combination of multi-source remote sensing data is an efficient way to achieve high-precision inversion of wetland vegetation biomass on a large scale. UAV imagery has ultra-high resolution but limited coverage, whereas satellite remote sensing data have wide spatial coverage and can provide abundant spectral information. For example, new hyperspectral images (e.g., GF-5, EnMap and PRISMA) contain hundreds of consecutive narrow bands, providing new possibilities for more accurate quantitative estimation of vegetation traits (Castaldi et al., 2016; Verrelst et al., 2021). The combination of multiplatform remote sensing data is powerful for dynamically monitor wetland vegetation with high application potential (Gao et al., 2019).

High-resolution remote sensing images provided by consumer-grade UAVs can realize high-precision wetland vegetation biomass inversion at the species level. Overcoming temporal and spatial limitations, this technique can assist in mapping biomass distribution, which is of great significance to the monitoring of invasive and dominant species, with great application prospects.

## 6 Conclusion

In this study, univariate regression models and the BPNN were compared to estimate the AGB of *Z. latifolia* in Honghu Wetland, demonstrating the feasibility of using UAV-based RGB images to monitor the growth status of wetland vegetation. The main conclusions are as follows:

(1) The AGB of *Z. latifolia* was significantly correlated with CIVE, COM2, ExG, G, ExGR, COM, R and B. The highest correlation was found with CIVE with an absolute correlation coefficient of 0.87. The vegetation index derived from the UAV RGB images can be used as an indicator for the AGB inversion of *Z. latifolia*.

(2) Among the univariate regression models constructed by CIVE, COM2 and ExG, the quadratic model based on CIVE has the highest inversion accuracy (validation R^2 ^= 0.37, RMSE=853.76 g/m^2^, MAE = 671.28 g/m^2^). The BPNN constructed with eight vegetation indices had the best inversion effect (validation R^2 ^= 0.68, RMSE=732.88 g/m^2^, MAE=583.18 g/m^2^). Compared with the quadratic model constructed by CIVE, the validation R^2^ was increased by 0.31, RMSE and MAE were reduced by 120.88 g/m^2^ and 88.10 g/m^2^, respectively. The results showed that the BPNN was the best model for the AGB inversion of *Z. latifolia* in Honghu Wetland.

(3) Although the spectral information of UAV-based RGB images is limited, their high-resolution can provide abundant features, which is helpful for the classification and biomass inversion of wetland vegetation at the species level. Consumer-grade UAVs are easier to deploy in complex wetland environments and have a distinct advantage in data acquisition. Future research should focus on the use of UAV images combined with satellite remote sensing images to monitor the growth of different types of wetland vegetation, and explore the relationship between non-destructive indicators and vegetation biomass.

## Data availability statement

The raw data supporting the conclusions of this article will be made available by the authors, without undue reservation.

## Author contributions

RZ, EL, and CY conceived the study, designed the experiments, and collected the data. RZ analyzed the data and completed the laboratory experiments. RZ, EL, and CY constructed the methodological framework and engaged in critical discussion. RZ wrote the manuscript. EL, CY, XC, and XW revised the manuscript. All authors contributed to the article and approved the submitted version.
